# Neural progenitor cells attenuate inflammatory reactivity and neuronal loss in an animal model of inflamed AD brain

**DOI:** 10.1186/1742-2094-6-39

**Published:** 2009-12-23

**Authors:** Jae K Ryu, Taesup Cho, Yu Tian Wang, James G McLarnon

**Affiliations:** 1 Department of Anesthesiology, Pharmacology and Therapeutics, University of British Columbia, Vancouver, British Columbia V6T 1Z3, Canada; 2 Brain Research Centre, Vancouver Coastal Health Research Institute, University of British Columbia, Vancouver, British Columbia V6T 1Z3, Canada

## Abstract

**Background:**

Transplantation of neural progenitor cells (NPC) constitutes a putative therapeutic maneuver for use in treatment of neurodegenerative diseases. At present, effects of NPC transplantation in Alzheimer's disease (AD) brain are largely unknown and a primary objective of this work was to demonstrate possible efficacy of NPC administration in an animal model of AD. The benefits of transplantation could involve a spectrum of effects including replacement of endogenous neurons or by conferring neuroprotection with enhancement of neurotrophic factors or diminishing levels of neurotoxic agents. Since chronic inflammation is a characteristic property of AD brain, we considered that transplantation of NPC could have particular utility in inhibiting ongoing inflammatory reactivity. We have tested intrahippocampal transplantation of NPC for efficacy in attenuating inflammatory responses and for neuroprotection in beta-amyloid (Aβ_1-42_) peptide-injected rat hippocampus.

**Methods:**

Spheres of neural progenitor cells were grown from dissociated telencephalon tissue of rat embryos. NPC were infected with lentiviral vector green fluorescent protein (GFP) with subsequent cell transplantation into rat hippocampus previously injected (3 d prior) with Aβ_1-42 _peptide or PBS control. Immunohistochemical analysis was carried out (7 d post-NPC transplantation, 10 d post-peptide/PBS injection) for GFP, microgliosis (Iba-1 marker), astrogliosis (GFAP marker), neuron viability (MAP-2 marker) and levels of the proinflammatory cytokine, TNF-α.

**Results:**

Successful infection of cultured NPC with lentiviral vector green fluorescent protein (GFP) was demonstrated prior to cell transplantation into rat hippocampus. *In vivo*, immunohistochemical staining showed migration of GFP-positive cells, in a region of dentate gyrus between Aβ_1-42_/PBS injection site and NPC transplantation site, was increased ×2.8-fold with Aβ_1-42 _compared to PBS injection. Double immunostaining in peptide-injected brain indicated GFP association with nestin and GFAP, but not MAP-2. Cell-specific immunostaining showed marked increases in microgliosis and astrogliosis in Aβ_1-42_-injected brain (respective increases of ×4.3- and ×4.6-fold compared with PBS injection). NPC transplantation significantly reduced microgliosis (by 38%) but not astrogliosis in peptide-injected hippocampus. The proinflammatory cytokine TNF-α was elevated by 6.7-fold (peptide vs PBS injection) with NPC administration attenuating levels of TNF-α (by 40%). Peptide-injected brain demonstrated neuronal loss (MAP-2 staining reduced by 45% vs PBS injection) with NPC transplantation effective in conferring neuroprotection (26% recovery of neurons).

**Conclusions:**

These findings indicate efficacy for NPC transplantation in an animal model of AD with effects consistent with cellular actions to attenuate inflammatory reactivity induced by intrahippocampal peptide injection.

## Background

Alzheimer's disease (AD) is a chronic neurodegenerative disorder that in advanced stages is characterized by increased levels of amyloid-beta (Aβ) peptide deposits, neurofibrillary tangles, abnormalities in neuronal and synaptic function and evidence for ongoing inflammatory reactivity [1,2]. The changes in underlying brain processes are manifest in a marked deterioration in memory and cognition. Numerous risk factors such as aging are associated with, and exacerbate, the loss of function in AD brain [3]. Importantly, the multiple processes and risk factors contributing to the slow progression of AD pathology compromise therapeutic strategies for treatment of the disease.

Transplantation of neural stem cells (NSC) constitutes a putative therapeutic maneuver for cell replacement in brain damage due to their intrinsic properties of self-renewal and capability for differentiation into different cell types including neurons. However, evidence also suggests stem cell therapy may confer neuroprotection by means other than cell replacement including the enhancement of neurotrophic factors [4] or by diminishing levels of putative neurotoxic factors. In the latter case, recent work has indicated efficacy of NPC may involve inhibition of inflammatory factors and responses [5,6]. Overall, beneficial effects of stem cell administration have been reported in a number of animal models including multiple sclerosis [7], Parkinson's disease [8] and stroke [9]. A recent study [10] has provided the first report for use of stem cell therapy in AD with the finding that transplantation improved cognitive performance in transgenic mice by elevation of brain-derived neurotrophic factor (BDNF).

Since chronic inflammation is a critical facet of AD brain, we reasoned that transplantation of neural progenitors could serve as a feasible strategy to attenuate ongoing inflammatory reactivity and thereby protect neurons. Furthermore, capacity for neural progenitors to engage in chemotactic activity has recently been reported [11,12], a necessary requirement for increased mobility in response to inflammatory factors. We have tested this hypothesis by measuring migration of transplanted neural progenitor cell (NPC) and effects of NPC transplantation on inflammatory responses mediated by microglia and astrocytes, levels of the proinflammatory cytokine, TNF-α and neuronal viability in an animal model of inflamed AD brain. This model uses intrahippocampal injection of amyloid-beta peptide (Aβ_1-42_) to induce marked inflammatory reactivity with concomitant neuronal damage in rat brain [13,14].

## Methods

### Neurosphere cultures

Spheres of neural progenitor cells were grown from dissociated telencephalon tissue of 14 d Sprague-Dawley rat embryos in neurobasal medium (GIBCO) containing B27 (GIBCO) supplement with 20 ng/ml basic fibroblast growth factor (bFGF) (PeproTech), 10 ng/ml epidermal growth factor (EGF) (PeproTech) and 10 ng/ml leukemia inhibitory growth factor (LIF) (Chemicon). The procedure of changing culture medium every 3 days results in the formation of neurospheres [15].

### Immunostaining of neurospheres

Neurospheres were plated on 12 mm round cover glass (Deckglaser). Spheres were fixed in 4% paraformaldehyde for 10 min, permeabilized in 0.1% Triton X-100 (Sigma) in PBS for 5 min, blocked in 5% normal goat serum (NGS) in phosphate buffered saline (PBS) for 1 hour, and incubated at 4°C overnight with the following primary antibodies: anti-nestin (1:200; Chemicon), anti-vimentin (1:500; Sigma), anti-microtubule associated protein-2 (MAP-2) (1:1000; Chemicon), anti-glial fibrillary acidic protein (GFAP) (1:100; Sigma), and anti-green fluorescent protein (GFP) (1:1000; Invitrogen). The spheres were subsequently incubated with anti-mouse and anti-rabbit secondary antibodies conjugated to Alexa Fluor 488 and 555 (IgG, 1:1000; Molecular probes) for 1 hour at room temperature. The spheres were then incubated in 4',6-diamidino-2-phenylindole dihydrochloride (DAPI) (1:1000; Sigma) for 30 sec and coverslipped in polyvinyl alcohol mounting medium with DABCO-antifade solution (Sigma). Control immunostaining was performed by omission of the primary antibody. Fluorescent images were obtained from a Leica DMIRE2 deconvolution microscope using the software OpenLab 3.7.

### Stereotaxic injection of fibrillar Aβ_1-42_

All experimental procedures were approved by the University of British Columbia Animal Care Ethics Committee, adhering to guidelines of the Canadian Council on Animal Care. Full-length peptide (Aβ_1-42_; California Peptide) was prepared as previously described [16,17]. The compounds were first dissolved in 35% acetonitrile (Sigma) and further diluted to 500 μM with incremental additions of PBS with vortexing. The peptide solution was subsequently incubated at 37°C for 18 hr to promote fibrilization and aggregation and stored at -20°C. Intrahippocampal injection of Aβ_1-42 _was performed as previously described [13,14]. In brief, male Sprague-Dawley rats (Charles River) weighing 280-300 g were anesthetized (ketamine/xylazine, i.p.) and placed in a stereotaxic apparatus (David Kopf Instruments, Tujunga, CA) and received unilateral injection of 2 nmol Aβ_1-42 _at the following coordinates: anteriorposterior (AP): -3.3 mm, mediallateral (ML): -1.6 mm, (dorsoventral) DV: -3.6 mm, from bregma [18]. Control animals received injection of PBS at these coordinates.

### Transplantation of GFP labelled neural progenitor cells

Dissociated neural progenitor cells in Hank's balanced salt solution were transduced with lentiviral vectors carrying an enhanced green fluorescent protein (pHR'-CMV-GFP). The efficiency of GFP expression levels was quantified in vitro. Approximately 5 × 10^5 ^NPCs were seeded and infected with 3-fold higher titre lentivirus (compared with seeded cell density) in 12 mm converslips *in vitro*. Three days after infection, NPCs were fixed with 4% PFA and placed under a fluorescence microscope for GFP measurement: the results showed approximately 40% of NPCs expressed GFP expression.

The transduced neural progenitor cells (NPC-GFP; 5 × 10^4^, 3 μl) were then stereotactically transplanted (0.20 μl/min) into the hippocampus. Site of transplantation was chosen close to the peptide injection site at the following coordinates from bregma (AP: -3.3 mm, ML: -1.8 mm, DV: -3.2 mm) as previously described [19]. For control cell graft, dead NPC were prepared by repeated cycles of freezing and thawing and used as control graft [20]. Transplantation was performed three days after PBS and Aβ_1-42_injection. Immunosuppressive agents were not used in the transplantation protocols due to the possibility of anti-inflammatory effects of the agents that could complicate immune-modulatory actions of NPC *in vivo*.

### Immunohistochemical analysis

Seven days after NPC transplantation, rats were anesthetized and killed by transcardiac perfusion of saline, followed by 4% paraformaldehyde. Brains were then removed, post-fixed, cryoprotected, and sectioned into 40 μm throughout the hippocampus [14]. Free-floating sections were processed for immunohistochemistry as described previously [14]. Briefly, sections were permeabilized in 0.2% Triton X-100, blocked with 10% NGS, and incubated overnight at 4°C with the primary antibodies: anti-GFP (1:1000; Invitrogen), anti-ionized calcium-binding adapter molecule 1 (Iba-1, 1:1000; Wako Chemicals), anti-GFAP (1:1000; Sigma), anti- tumor necrosis factor-alpha (TNF-α, 1:200; Cedarlane Laboratories Ltd), and anti-MAP-2 (1:500; Sigma). Sections were incubated with secondary antibodies for 1 hour at room temperature, mounted on Superfrost/Plus microscope slides (Fisher Scientific), and coverslipped. For immunostaining controls, primary antibodies were omitted from the staining procedures. For double immunofluorescence staining, free-floating sections were incubated overnight at 4°C with primary antibody to GFP (1:1000; Invitrogen) with nestin (1:500; Chemicon), GFAP (1:1000; Sigma), or MAP-2 (1:500; Sigma) and incubated for 1 hr with a mixture of Alexa Fluor-conjugated 488 anti-rabbit IgG (1:100; Molecular Probes) and Alexa Fluor 594-conjugated anti-mouse IgG (1:100; Molecular Probes). Immunofluorescence images were examined under a Zeiss Axioplan 2 fluorescent microscope (Zeiss) using a DVC camera (Diagnostic Instruments) with Northern Eclipse software (Empix Imaging) and analyzed for colocalization of staining using National Institutes of Health Image J.

### Cell-associated immunostaining

The extents of microgliosis (Iba-1 marker), astrogliosis (GFAP marker) and TNF-α immunoreactivity (ir) induced by intrahippocampal Aβ_1-42 _or control PBS injections, were evaluated by measuring the marker pixel intensities from five hippocampal sections [14,21]. Immunostaining was done over the specific areas of dentate gyrus, molecular layer (ML) and granule cell layer (GCL). The immunostained section images were digitized and analyzed using the image analysis program NIH version 1.57 (Wayne Rasband, NIH). The overall neuronal viability was assessed by measuring the ir of MAP-2 staining in the ML and GCL of the hippocampus. All quantitative analyses were performed in a blinded manner.

### Statistical analysis

All data are expressed as means ± SEM. Statistical significance of differences for group comparisons was assessed using analysis of variance followed by Bonferroni's post hoc test or Student's *t *test. Significance was set at *p *< 0.05.

## Results

### Patterns of distribution and differentiation of transplantedNPC, in vivo

**Figure 1:**
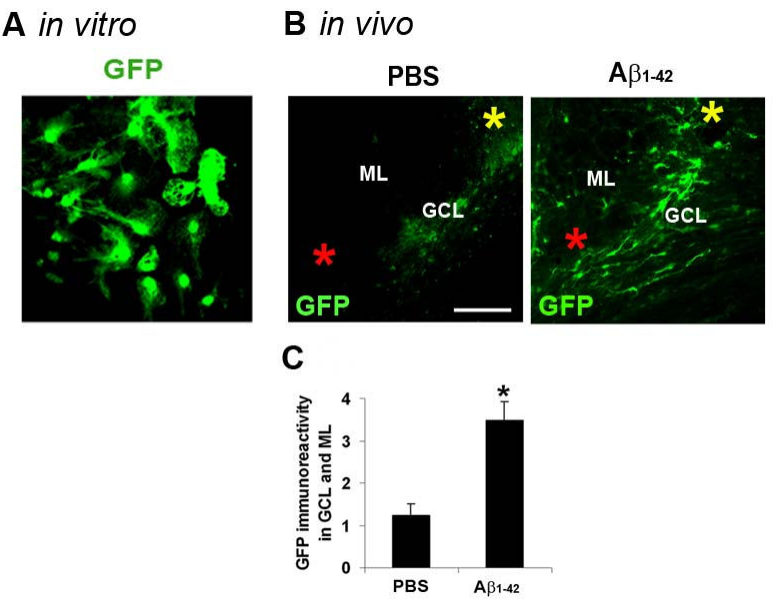
**GFP-labelled NPC *in vitro *and diffusion of GFP (+)ve NPC *in vivo***. (A) GFP staining of cultured NPC. (B) Representative immunostaining for GFP (+)ve NPC in molecular (ML) and granule cell (GCL) layers of dentate gyrus for PBS and Aβ_1-42_-injected rat brain, scale bar is for 100 μm. The site of PBS or Aβ_1-42 _injection is indicated by a red asterisk; the location of NPC transplantation is indicated by a yellow asterisk. (C) Quantification of GFP ir in region between site of injection (Aβ_1-42 _or PBS) and site of NPC transplantation (N = 4 animals/group, * denotes p < 0.05).

Initial studies demonstrated that cultured NPC expressed characteristic markers for undifferentiated stem cells including nestin and vimentin; NPC also expressed the astrocytic marker, GFAP but not neuronal MAP-2 (data not shown). As shown in Fig. 1A, successful transduction of lentiviral vector-GFP was demonstrated in cultured NPC prior to cell intrahippocampal transplantation. Transplantation of GFP-labeled NPC into the dentate gyrus was carried out 3 days subsequent to intrahippocampal injections of control PBS or Aβ_1-42 _(at 2 nmol). Immunohistochemical analysis was carried out at 7 d following NPC transplantation (10 d post-Aβ_1-42_/PBS injection). Representative GFP immunostaining, in the molecular and granule cell layers (ML and GCL), indicated increased numbers of NPC in the vicinity of Aβ_1-42 _(right panel, Fig. 1B), compared with PBS (left panel, Fig. 1B), injection site. In order to assess dispersion and net migration of NPC from the site of transplantation, immunoreactivity (ir) of GFP (+)ve cells was measured in regions between the sites of peptide/PBS injection and NPC transplantation. The results (Fig. 1C) demonstrated considerably increased GFP ir in these areas in Aβ_1-42_-injected, relative to PBS-injected, hippocampus. Overall, area density of GFP ir was increased by ×2.8-fold with peptide, compared with PBS, injection.

**Figure 2:**
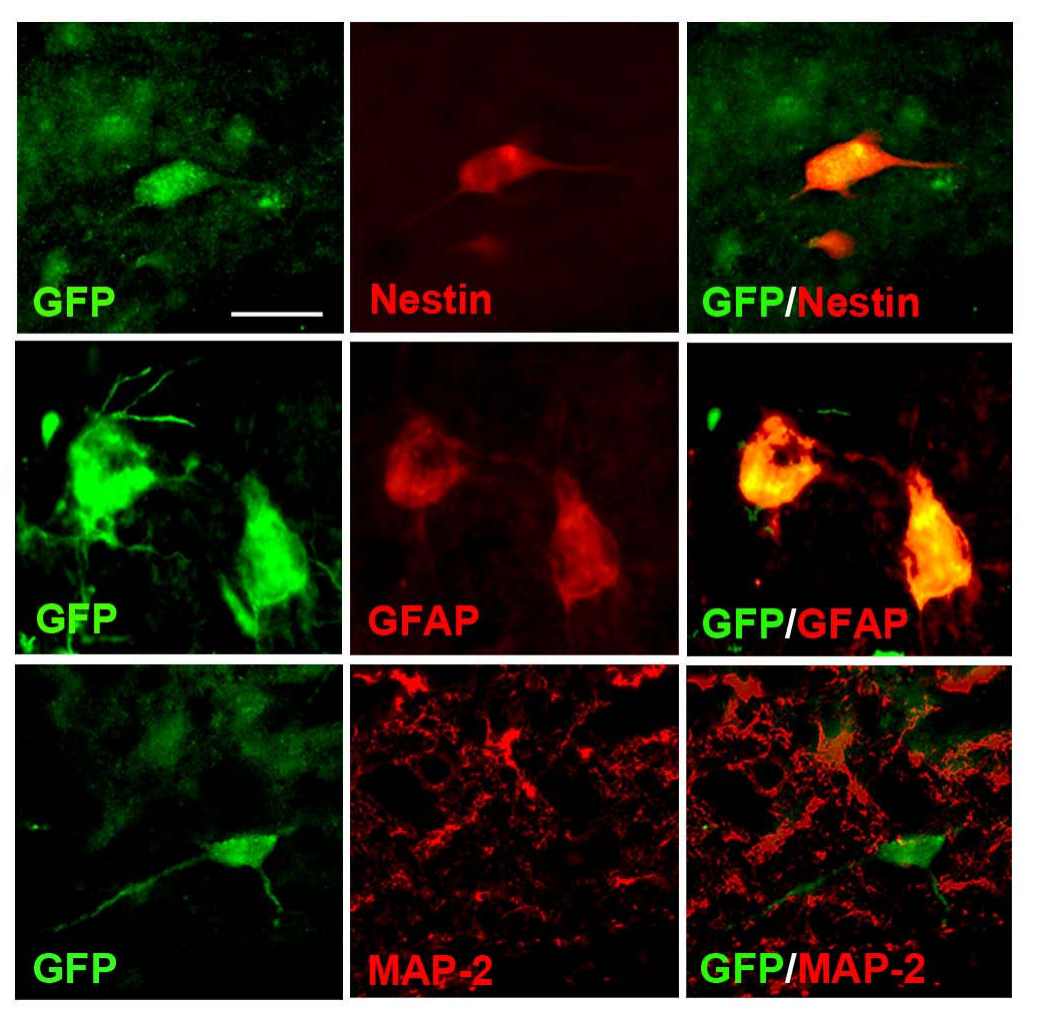
**Expression of markers in injected NPC**. Representative double staining for GFP association with nestin (upper panel), GFAP (middle panel) and MAP-2 (bottom panel), scale bar = 10 μm.

Undifferentiated NPC exhibit a number of cell-specific properties and markers such as nestin. We examined for expression of characteristic properties of transplanted cells *in vivo *in peptide-injected hippocampus. Representative staining patterns of GFP with the different cellular markers (nestin, GFAP and MAP-2) are presented in Fig. 2. Results from double-immunostaining analysis demonstrated GFP-labeled cells to express nestin and GFAP (Fig. 2, upper and middle panels). Overall, we found in excess of 90% of NPC showed expression of both nestin and GFAP. However, no evidence for MAP-2 colocalization with GFP was found in Aβ_1-42_-injected hippocampus (Fig. 2, lower panels). The lack of MAP-2 association with GFP ir suggests that with short term transplantation little or no NPC differentiated into neurons.

### Effect of NPC on A_β1-42_-induced inflammatory reactivity

The results shown in Fig 1 indicate NPC migration in response to intrahippocampal Aβ_1-42_-injection. Since microgliosis and astrogliosis are upregulated after peptide injection, gliosis could be modulated in the presence of NPC grafting. To examine this point, the effects of 7 d NPC transplantation on microglial and astrocyte inflammatory responses and levels of the proinflammatory cytokine, TNF-α were examined in five animal groups; Aβ_1-42 _or PBS injected rat hippocampus, Aβ_1-42 _plus NPC, Aβ_1-42 _plus dead NPC and NPC alone; PBS served as a control for peptide injection and dead NPC were used as a control for NPC.

**Figure 3:**
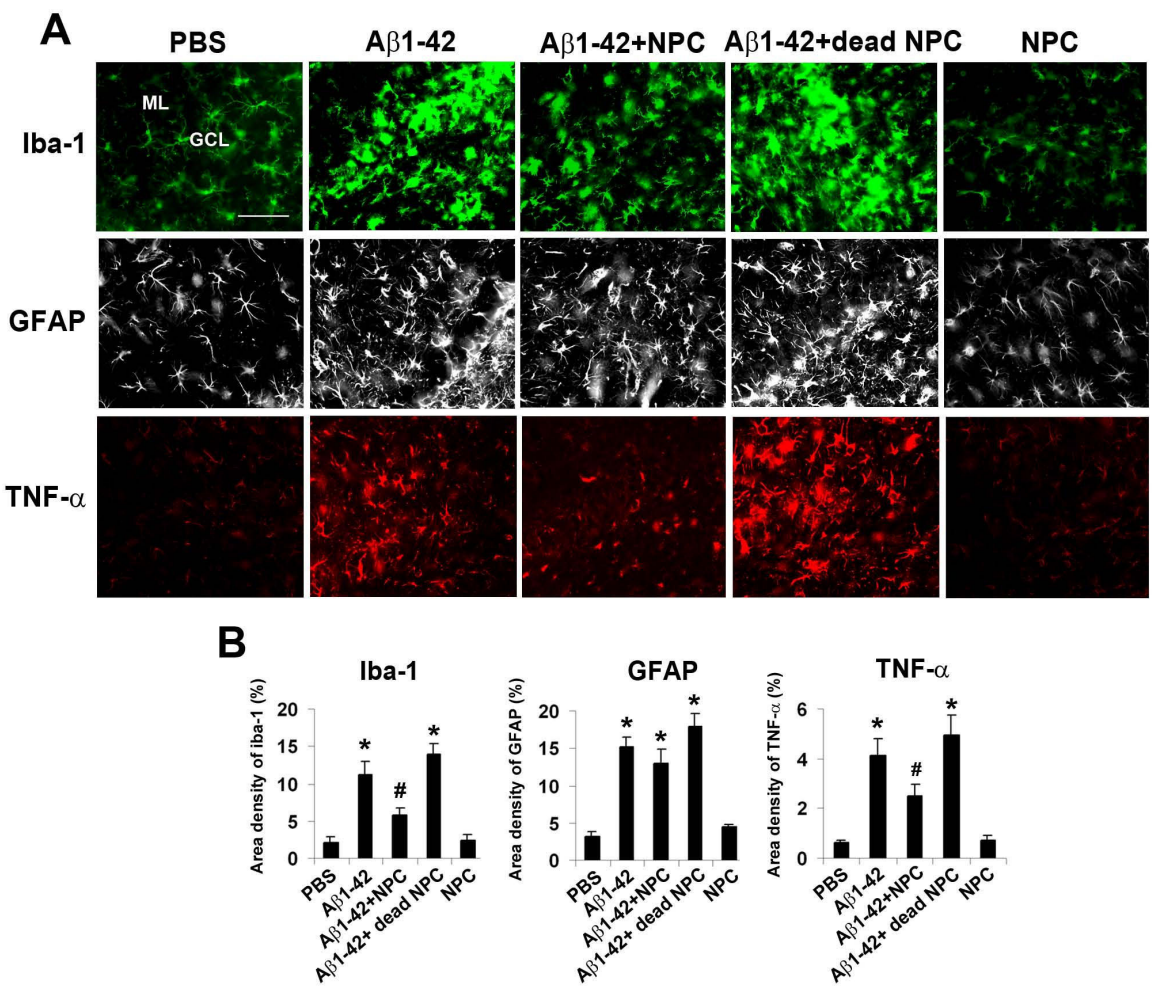
**Effects of NPC transplantation on inflammatory reactivity**. (A) Representative staining for microglial responses (Iba-1 marker, upper panels), astroglial responses (GFAP marker, middle panels) and levels of TNF-α (lower panels) in GCL and ML following 10 d intrahippocampal injections of PBS/Aβ_1-42 _and 7 d transplantation of NPC/dead NPC. The animal groups (panels, left to right) are for PBS, Aβ_1-42_, Aβ_1-42 _+ NPC, Aβ_1-42 _+ dead NPC and NPC alone, scale bar represents 100 μm. (B) Quantification of data for Iba-1 (left bar graph), GFAP (middle bar graph) and TNF-α (right bar graph) for the different animal groups (N = 5 animals/group, * denotes p < 0.05 compared with PBS, # denotes p < 0.05 compared with Aβ_1-42_).

Representative immunostaining for microglia (Iba-1 marker), localized to areas between injection (Aβ_1-42 _or PBS) sites and NPC transplantation site, is shown for the different experimental groups (10 d post-Aβ_1-42_/PBS injection) in Fig. 3A (upper panels). Peptide-injected brain demonstrated a considerably elevated Iba-1 ir compared with PBS-injection. NPC transplantation in Aβ_1-42_-injected animals showed efficacy in reducing extents of Iba-1 ir, however, transplantation of dead NPC with peptide was ineffective in reducing microglial proliferative responses. NPC transplantation alone showed a pattern of Iba-1 ir similar to PBS control. Quantification of data is presented in Fig. 3B (left bar graph). Overall, microgliosis (measured as area density of Iba-1 ir in ML/GCL) was increased ×4.3-fold in Aβ_1-42_, relative to PBS, injected brain. Transplantation of NPC in peptide-injected animals significantly reduced microgliosis (by 38%) compared with Aβ_1-42_-injected animals receiving no transplantation. Levels of Iba-1 ir were not significantly altered with application of dead NPC in peptide-injected animals. NPC transplanted animals, in the absence of peptide administration, showed low extents of microgliosis.

Representative immunofluorescent staining for GFAP, in the same regions used for analysis of microgliosis, is shown in Fig. 3A (middle panels) for the different animal groups. Peptide-injected brain exhibited an increased GFAP ir relative to PBS control. Interestingly, levels of astrogliosis appeared relatively unchanged in Aβ_1-42_-injected rats receiving NPC transplantation. GFAP ir in animals receiving transplantation alone was similar to marker ir with PBS control injection. Quantification of data (Fig. 3B, middle bar graph) showed astrogliosis to be significantly increased (×4.6-fold) in peptide, relative to PBS, injected rat brain. Although a small decrease in GFAP ir was measured (14%) with NPC transplantation in peptide-injected brain, this effect was not significant. Extents of GFAP ir were not significantly different between animals receiving Aβ_1-42 _and Aβ_1-42 _+ dead NPC or between groups administered PBS injection and ones receiving NPC transplantation alone.

Expression of TNF-α was minimal in PBS-injected hippocampus with high expression of the cytokine evident in peptide-injected brain (Fig. 3A, lower left panels). Transplantation of NPC, but not dead cells, was highly effective in attenuating expression of TNF-α in peptide-injected hippocampus. NPC transplantation alone had no effect to alter levels of the cytokine compared to PBS control. Overall, expression of TNF-α in ML/GCL was increased ×6.7-fold in Aβ_1-42_, compared with PBS, injected hippocampus (Fig. 3B, right bar graph). Transplantation of NPC into peptide-injected brain significantly reduced levels of the pro-inflammatory cytokine, by 40%, compared with Aβ_1-42 _injection alone. No significant differences in TNF-α ir were measured between peptide and peptide plus dead NPC animal groups or between PBS injected, and NPC transplanted, brain.

### Effect of neural progenitors on Aβ_1-42_-induced neuronal injury

**Figure 4:**
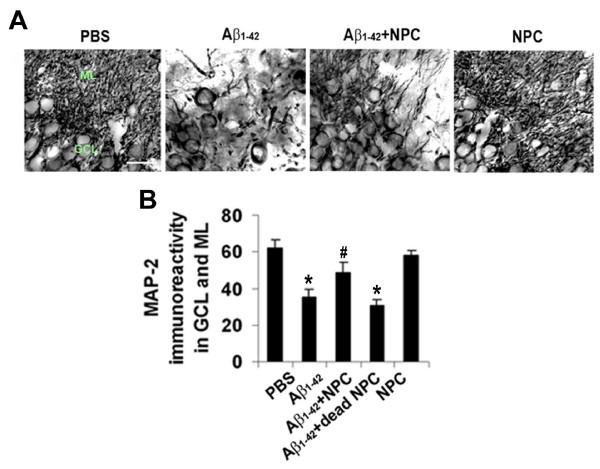
**Effects of NPC transplantation on neuronal viability**. (A) Representative high magnification of MAP-2 ir in GCL and ML, scale bar is for 20 μm. Animal groups (panels left to right) are for PBS, Aβ_1-42_, Aβ_1-42 _+ NPC and NPC alone. (B) The bar graph presents quantification of MAP-2 ir in GCL/ML (N = 5 animal groups, * denotes p < 0.05 vs. PBS, # p < 0.05 vs. Aβ_1-42_).

A critical objective of this work was to determine efficacy of NPC transplantation on neuronal viability. The region of study was the same as for assessment of NPC migration and gliosis, localized to areas between injection and transplantation sites. Representative high magnification patterns of immunostaining for neurons (MAP-2 marker) are presented for PBS and Aβ_1-42 _injected hippocampus (Fig. 4A). The results indicate a considerable loss of MAP-2 (+)ve neurons with Aβ_1-42_, compared to PBS, injection (Fig. 4A, left panels). NPC transplantation in peptide-injected animals (Fig. 4A, second panel from right) was effective in attenuating the loss of neurons. Dead NPC were ineffective when applied in peptide-injected brain (data not shown). The control NPC graft alone (Fig. 4A, right panel) presented a similar pattern of MAP-2 ir as found with PBS injection. Overall (N = 5 animals/group), MAP-2 ir in ML and GCL was diminished by 45% in Aβ_1-42_, relative to PBS, injected animals (Fig. 4B). Animals receiving NPC transplantation showed a significant (26%) increase in numbers of MAP-2 (+)ve neurons compared to peptide-injected animals not receiving NPC treatment. Levels of MAP-2 ir were not significantly different between Aβ_1-42 _alone and Aβ_1-42 _plus dead NPC or between PBS-injected and NPC-transplanted animals.

## Discussion

The primary objective of this study was to provide evidence for the potential clinical utility of NPC transplantation in AD brain. The major findings from the work are that NPC transplantation significantly inhibits inflammatory reactivity and provides neuroprotection in the Aβ_1-42_-injected rat hippocampus. The results constitute the second report of beneficial effects of stem cell treatment in AD; a recent study has demonstrated improvement in cognitive behaviour in transgenic animals with effects attributed to increased levels of hippocampal BDNF [10]. As discussed below, our data showing correlation between inflammatory reactivity and neuronal viability support the possibility that NPC actions to attenuate inflammatory responses may have utility in reducing neuronal damage in inflamed AD brain.

We found that cultured NPC, isolated from rat brain, exhibited a spectrum of characteristic features of undifferentiated stem cells including expressions of nestin, vimentin and GFAP. Efficient transduction of the cells with GFP was demonstrated for the cultured NPC prior to their *in vivo *transplantation into rat hippocampus at 3 d following intrahippocampal injections of PBS control or Aβ_1-42_. At 7 d following NPC transplantation (10 d after Aβ_1-42 _or PBS), immunohistochemical analysis showed higher dispersed GFP ir between sites of injection and transplantation with peptide, relative to PBS, injection. These results are consistent with Aβ_1-42 _injection stimulating a migration of NPC from transplantation to injection site. However, over the whole hippocampus we observed no evident differences between GFP ir with peptide or PBS injection suggesting that NPC survival was not a factor in our experiments. Double staining *in vivo *showed prominent immunoreactivity of GFP, colocalized with progenitor cell nestin and GFAP, suggesting a NPC phenotype as undifferentiated cells. No evidence for NPC neuronal differentiation was evident (marker MAP-2), a result which could reflect the relatively short duration of NPC grafting employed in this work.

We conclude migration of NPC from sites of transplantation to sites of injection was enhanced in peptide-injected, compared to PBS-injected, hippocampus. These findings would be consistent with the presence of chemotactic stimulatory signals which increase NPC mobility in peptide-injected brain. The initiating stimulus for induction of increased NPC migration could be due to direct deposition of Aβ_1-42 _or indirectly due to signals from microglia (see below) that have been activated by peptide. In the latter case, we have documented that injection of Aβ_1-42 _into dentate gyrus is a potent stimulus for induction of microglial chemotactic responses mediated by a specific receptor for vascular endothelial growth factor (VEGF) [21]. Interestingly, recent work has reported stromal cell derived factor-1 and its receptor CXCR4 as modulators of progenitor cell migration in the dentate gyrus [11,12,22].

Intrahippocampal injection of Aβ_1-42 _was associated with considerable increases in microgliosis and astrogliosis compared with PBS control. Transplantation of NPC after Aβ_1-42 _injection significantly inhibited microgliosis, but not astrogliosis, in proximity to peptide injection site. Microgliosis was not altered with dead NPC administered to peptide-injected hippocampus and NPC transplantation alone was associated with similar levels of gliosis as for PBS injection. Cytokine levels are enhanced in AD brain [23] and our results showed elevated TNF-α, a pro-inflammatory cytokine with autocrine function in microglia [24], in Aβ_1-42_-injected hippocampus. Administration of NPC, but not dead progenitor cells, attenuated levels of TNF-α. As discussed below, the effects of NPC grafting to inhibit microgliosis and levels of TNF-α may be correlated.

The injection of Aβ_1-42 _was associated with a loss of neuronal viability, compared with PBS control injection, consistent with previous findings [14,16,17]. Importantly, transplantation of NPC, but not dead cells, was effective in diminishing loss of neurons in peptide-injected brain. Although underlying neuroprotective mechanisms are not well understood, this result could be linked with the finding that NPC transplantation was efficacious in attenuating microgliosis with no significant actions to alter astrogliosis. One possibility to account for effects of NPC on microglial responses is that peptide-induced activation of microglia increases their production of chemokines including monocyte chemoattractant protein-1 (MCP-1) [25] and interleukin-8 (IL-8) [26]. In this event preferential migration of NPC to areas exhibiting microglial proliferative responses may follow. Increased migration of stem cells induced by microglia [27], and specifically by the factor MCP-1 [28], have been reported, *in vitro*. Subsequent NPC release of neurotrophic factors [10] could then inhibit microglial activation by blocking cell-specific inflammatory factors such as major histocompatibility class II [29]. Since Aβ-stimulated microglia are potent producers of TNF-α [24], NPC-mediated effects to decrease microglial activation would be consistent with the diminished levels of the pro-inflammatory cytokine as found following NPC grafting. Indeed, our results demonstrated very similar extents of reductions (about 40%) in microgliosis and levels of TNF-α with NPC transplantation. Previous studies have suggested that pharmacological maneuvers that inhibit microglial activation can attenuate neuronal damage in animal models of AD [13,21]. Overall, our findings are consistent with an enhanced migration of NPC in response to signals from peptide-activated microglia with NPC releasing factors which in turn act to inhibit microglial inflammatory reactivity. At present, however, the specific NPC-dependent factors coupled to reduction in inflammatory responses and neuroprotection have not been determined.

Our results, together with those reported in [10], provide a proof of principle that stem cell therapy could be efficacious in AD. A number of questions need to be addressed in AD animal models including the nature of microglial signals which mediate NPC migration and NPC-derived factors which modify microglial activation and inflammatory responses. The production and release of growth factors other than BDNF [10] by NPC could also contribute to increased neuronal viability and enhanced cognition. Another unresolved question is the possibility that NPC could also directly differentiate into functional neurons in diseased brain suggesting the utility of future work in using longer durations of NPC transplantation in AD animal models.

## Conclusions

It must be noted that any benefits in applying stem cell therapy as a treatment in AD are confounded by a number of complex issues including the involvement of multiple factors in disease pathology and the presumed loss of neuronal and synaptic viability in widespread regions of affected brains. Nevertheless, our data are noteworthy in demonstrating neuroprotective efficacy for NPC in an animal model of AD which likely emphasizes effects of inflammatory activity [30]. Although effects of NPC transplantation to enhance neuronal viability were modest, it is reasonable to assume that increased levels of neuroprotection could be conferred with different transplantation protocols including use of longer times and higher doses of NPC. Overall, our findings taken in association with recent work in transgenic mice [10], suggest that NPC transplantation represents a novel and plausible approach warranting extensive testing in AD animal models.

## Competing interests

The authors declare that they have no competing interests.

## Authors' contributions

JKR and TC equally carried out experiments and analysis of data. JGM and JKR conceived and designed experiments. JGM and YTW drafted and finalized the manuscript. All of the authors have read and approved the final manuscript.
